# Surface-Enhanced Raman Spectroscopy of the Endothelial Cell Membrane

**DOI:** 10.1371/journal.pone.0106283

**Published:** 2014-09-04

**Authors:** Simon W. Fogarty, Imran I. Patel, Francis L. Martin, Nigel J. Fullwood

**Affiliations:** 1 Division of Biomedical and Life Sciences, Lancaster University, Lancaster, Lancashire, United Kingdom; 2 Centre for Biophotonics, Lancaster Environment Centre, Lancaster University, Lancaster, Lancashire, United Kingdom; 3 Cavendish Laboratory, University of Cambridge, Cambridge, Cambridgeshire, United Kingdom; RMIT University, Australia

## Abstract

We applied surface-enhanced Raman spectroscopy (SERS) to cationic gold-labeled endothelial cells to derive SERS-enhanced spectra of the bimolecular makeup of the plasma membrane. A two-step protocol with cationic charged gold nanoparticles followed by silver-intensification to generate silver nanoparticles on the cell surface was employed. This protocol of post-labelling silver-intensification facilitates the collection of SERS-enhanced spectra from the cell membrane without contribution from conjugated antibodies or other molecules. This approach generated a 100-fold SERS-enhancement of the spectral signal. The SERS spectra exhibited many vibrational peaks that can be assigned to components of the cell membrane. We were able to carry out spectral mapping using some of the enhanced wavenumbers. Significantly, the spectral maps suggest the distribution of some membrane components are was not evenly distributed over the cells plasma membrane. These results provide some possible evidence for the existence of lipid rafts in the plasma membrane and show that SERS has great potential for the study and characterization of cell surfaces.

## Introduction

Colloidal gold particles, 5–20 nm in diameter, have been used for several decades in electron microscopy [1]. Their high atomic weight makes them easy to visualize using the transmission electron microscope. This method can also be used with the light or scanning electron microscope (SEM), but in this case a silver-intensification procedure is usually employed to make them visible because of the lower spatial resolution of the SEM and the gold nanoparticles act as nuclei to reduce silver ions to metallic silver spheres in the silver-intensification solution [1], [2]. The diameter of the silver particles can be regulated by the duration of the silver-intensification procedure [2], [3]. Silver-intensification technology is now used for the detection of epitopes in a wide variety of immunoassay techniques [4]–[6].

More recently, metallic nanoparticles have found new applications in the field of biomedicine [7], [8] and especially in the field of biospectroscopy [9]. There are advantages of biospectroscopy compared to conventional immunolabelling, which only provides information on a single epitope within a cell. Unlike immunolabelling, biospectroscopy has the potential to simultaneously detect all the biomolecules within a sample [10]–[12]. This includes proteins, lipids, carbohydrates and nucleic acids, giving a unique spectral “fingerprint” for each sample. The sensitivity of these biospectroscopy techniques can be significantly enhanced in Raman spectroscopy by the use of metallic nanoparticles. This is due to an effect called surface-enhanced Raman spectroscopy (SERS) [13]. This is a complex process with three main factors contributing to the SERS-enhancement effect [14]. Put very simply, the increases in sensitivity are due to the excitation of plasmons from the metal nanoparticles [15] generating a greatly enhanced signal from the sample. In some circumstances, the signal may be amplified by many orders of magnitude [16] with enhancements in the range of 10^8^ to 10^12^. Although this level of SERS-enhancement is not seen on biological samples, enhancement by one or two orders of magnitude is possible. The level of SERS-enhancement obtained is known to be influenced by several factors. These include the shape of the nanoparticles [17]–[20], and their density [21] as well as the excitation wavelength [22].

Most of the research to date with SERS uses nanoparticles which have been attached to reporter molecules. In these instances, the metallic nanoparticle has a small molecule attached to it; the signal from this molecule is greatly enhanced by the SERS effect and therefore can be detected at a much lower concentration than would otherwise be possible. Thus it is possible to detect the presence of reporter molecules at much lower levels than would be possible without the nanoparticle. This approach has been recently used to directly monitor real time events such as apoptosis in live cells [9]. Recent work has shown that it is also possible to use metallic nanoparticles without reporter molecules, to obtain enhanced spectra from cells [3]. This work showed that it was possible to obtain a 40-fold increase in signal-enhancement. Furthermore, since the maximum enhancement effect was estimated to be limited to ∼10 nm [23], this method provided information on all of the biomolecules in the immediate locality of the nanoparticle, *i.e.*, the molecular composition of the cell surface. This is information that is difficult to obtain in any other way.

**Figure 1:**
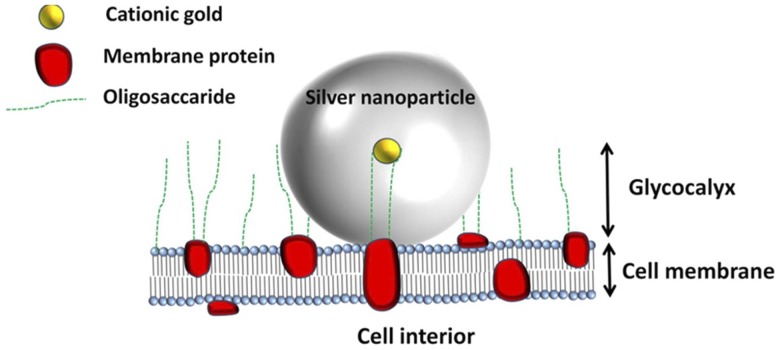
A schematic diagram of the silver-intensified cationic gold nanoparticle on the surface of the cell. The 15 nm cationic gold particle is shown interacting with the cell glycocalyx molecules.

Previous work has used gold-conjugated antibodies to attach the gold nanoparticles to the cell surface. However, there is not always a suitable cell surface antigen present, and there is also some debate as to the contribution of the antibody to the SERS-enhanced spectra. It is possible to attach nanoparticles to the cell surface using charge rather than an antibody/antigen interaction. Cationic gold will bind spontaneously to the negatively-charged glycocalyx [24], [25] on the cell surface and provides a much more even distribution of nanoparticles than can be achieved using antibody-conjugated methods. The glycocalyx on endothelial cells consists of glycolipids and glycoproteins with long oligosaccaride chains, extending several hundred nanometers from the cell surface (Figure 1).

This paper describes how cationic gold, followed by silver-intensification, can be used to enhance Raman spectra derived from the cell surface and thus obtain biomolecular information regarding the cell membrane. A schematic diagram of the protocol is shown in Figure 1. The cells used in this study are the corneal endothelium. The corneal endothelial cells form a remarkably flat and regular monolayer on Descemet's membrane on the inner surface of the cornea where the apical surface of the endothelial cells is readily accessible for the cationic gold.

## Methods

### Corneal samples

Fresh bovine corneas were obtained from a local abattoir (G &GB Hewitt Ltd, Cheshire, UK) within 2 hours of death and transported to the laboratory on ice. They were immediately dissected out and fixed using a 4% glutaraldehyde in phosphate-buffered saline (PBS) solution.

### Cationic colloidal gold-labelling

Cationic colloidal gold (15 nm) and identical silver-intensification kits were obtained from TAAB Laboratories (Berkshire, UK) or from BBI International (Cardiff, UK). The colloidal cationic gold was used as a stock solution and for this experiment was diluted using PBS to 1/10 and 1/100 concentrations.

The corneal samples were initially washed in PBS employing 3 washes of 5 minutes each. They were then incubated with one of the cationic colloidal gold dilutions at room temperature for 1 hour. The control samples were incubated with PBS instead of the cationic colloidal gold. Subsequently, all samples were washed in distilled water for 3 washes of 5 minutes each in order to remove any excess unbound substrate.

### Silver-intensification

The silver-intensification was carried out at room temperature. The silver enhancement was performed for 20 minutes during which the samples were kept away from bright illumination in order to limit the effect it would have on the enhancement process. The cationic gold treated samples which were not silver-intensified were incubated in PBS for 20 minutes.

Next, all the samples were washed with distilled water in 5 washes of 5 minutes each to ensure any unbound silver was washed off the samples. In preparation for analysis, the samples were dehydrated through an alcohol series by placing them in 50% ethanol for 5 minutes, followed by 70% ethanol for 10 minutes, 80% ethanol for 10 minutes, 90% ethanol for 10 minutes and, finally, washed 3 times in 100% ethanol for 10 minutes each before being air dried.

### Raman spectroscopy

Raman spectroscopy was carried out using a Renishaw InVia Raman machine with a 785 nm excitation laser (Renishaw Inc., UK), which was initially calibrated to 520.5 cm^−1^ with a silicon calibration sample before analysing the samples. After calibration, the prepared corneal samples were placed upon MirrIR Low-E glass slides (Kevley Technologies, USA) before being examined in order to allow an increase in the Raman signal gathered. All the Raman spectra were measured at 50x magnification down the microscope within the 500–2000 cm^−1^ spectral range using 1% laser power (0.1 mW), a 10 second accumulation time and 4 accumulations over the target illumination area of 1 µm^2^. An 830 l/mm grating was also used for each spectrum taken. For each different class, 40 spectra were taken with 20 taken from each sample in each class and the data was compiled in WiRE software (Renishaw Inc.). Some peaks were artefacts caused by ambient lighting; these wavenumber peaks were excised from the spectra.

### Scanning electron microscopy

Separate bovine cornea samples were prepared as described above and were finally dehydrated using HMDS. After the samples were dry, they were affixed to aluminium stubs and sputter-coated with gold before being analysed with a JEOL 5600 scanning electron microscope (JEOL, Tokyo, Japan).

### Multivariate data analysis

The spectral data that was gathered was processed initially in OPUS software (Bruker Optik GmbH, Germany), whereupon the spectra underwent rubberband baseline correction and vector normalization. After completion, the processed data underwent multivariate computational analysis using MATLAB software (Mathworks Inc, Natick, USA) using the IRootLab MATLAB toolbox [26]. Principal component analysis (PCA) was performed initially and the output from this was inputted into subsequent linear discriminant analysis (LDA).

### Raman Imaging

Raman map images were taken of individual endothelial cells using a 50x objective (NA = 0.75) and a 100 nm precision motorized stage (Renishaw Inc.). Spectral points were taken in a raster pattern and were taken with 1 accumulation for each point at 0.1% laser power and 10 seconds acquisition time over a target area of 0.8×0.8 µm^2^. The spectral range of the Raman map was 500–2000 cm^−1^, using a static grating; in total, 960 spectral points were collected over an 11 hour period. Wire software (Renishaw Inc.) was then used to generate false color images of the spectral map at discrete wavenumber peaks, using different colors to represent different intensities of Raman signal.

## Results

**Figure 2:**
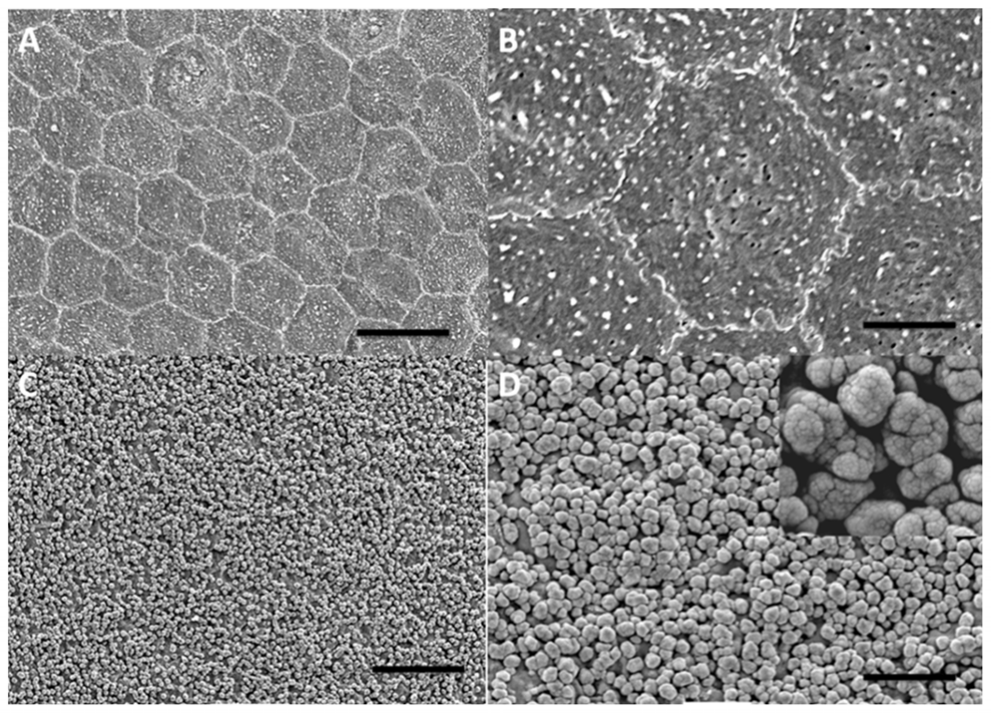
Scanning electron micrographs of the apical surface of endothelial cells. Corneal endothelium (**A, B**). Endothelium with 1/10 silver-intensified cationic gold (**C**). Endothelium with 1/10 silver-intensified cationic gold at a high magnification (**D**). (**A**) Scale bar  = 5 µm; (**B**) Scale bar  = 5 µm; (**C**) Scale bar  = 10 µm; and (**D**) Scale bar  = 5 µm. Insert ×5.

**Figure 3:**
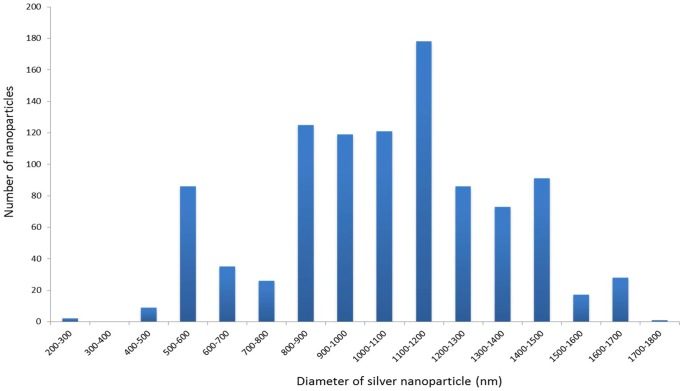
Histogram showing the range of diameters of silver-intensified cationic gold nanoparticles on the corneal endothelial cell surface.

The SEM results are shown in Figure 2. In Figure 2A and 2B, the characteristic hexagonal shape of the endothelial cells can be clearly seen. In Figure 2C and 2D, the silver-intensified gold nanoparticles are easily resolved on the cell surfaces. It is clear that there are a range of sizes and that there is evidence of closely associated nanoparticles or dimers. At very high magnification structures (2D insert) nano-crevices and nano-cracks are evident on the nanoparticles. Figure 3 shows a histogram of silver-intensified gold nanoparticle sizes. This confirms quantitatively the range of nanoparticle sizes with the modal size around 1100 nm.

**Figure 4:**
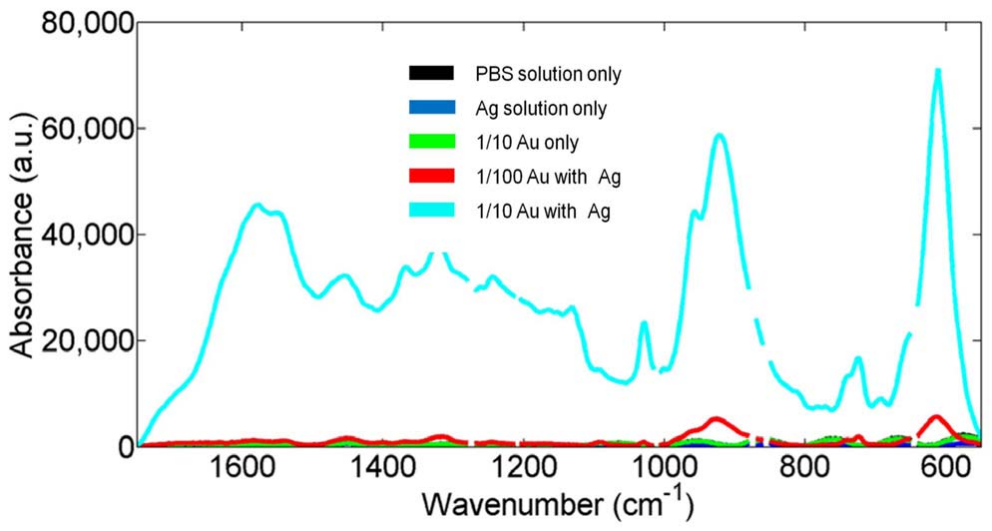
Mean spectra from the five different classes of samples; the spectra have been baseline corrected. The different treatments consist of: 1/10 silver-intensified gold (1/10 Au with Ag); 1/100 silver-intensified gold (1/100 Au with Ag); 1/10 gold without silver-intensification (1/10 Au only); silver-intensification solution without gold (Ag solution only) and PBS solution (PBS treatment only).

In Figure 4, the mean spectrum of each of the treatment groups is shown. The highest degree of SERS-enhancement was obtained with a 1/10 concentration of silver-intensified gold nanoparticles. This provides an approximate 100-fold degree of SERS-enhancement compared to the control. The 1/100 silver-intensified gold nanoparticle samples showed only a low level of SERS-enhancement in comparison (approximately 3-fold SERS-enhancement). The samples treated with cationic gold without silver-intensification or treated with silver-intensification solution alone appear identical to the PBS treated controls.

**Figure 5:**
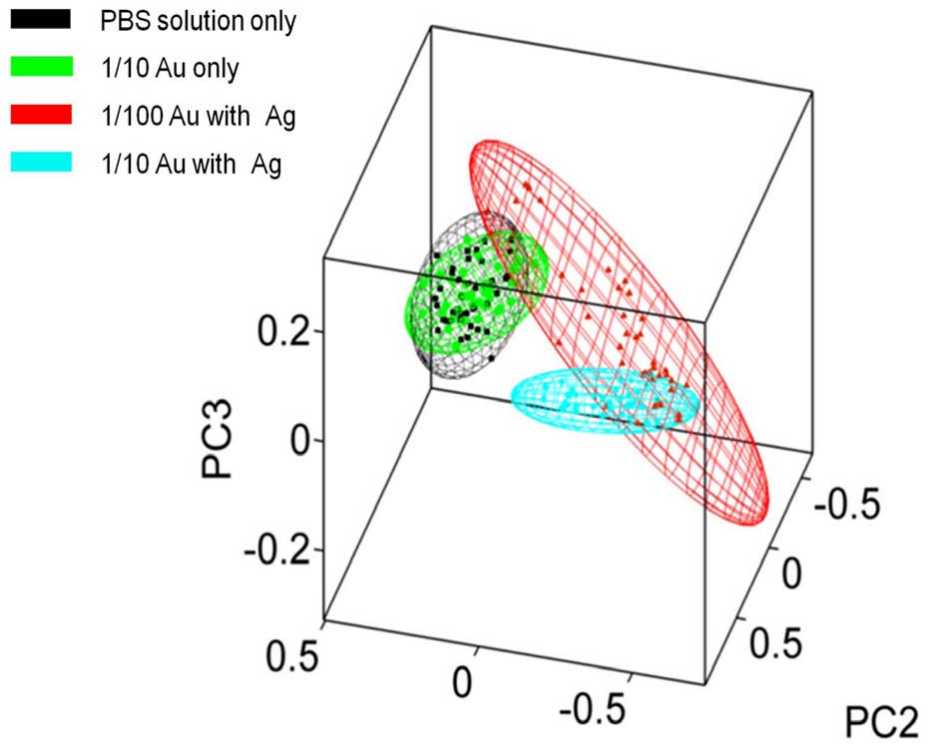
Three-D cluster plot of four different sample classes with 95% confidence ellipsoids. The classes consist of: 1/10 silver-intensified gold (1/10 Au with Ag); 1/100 silver-intensified gold (1/100 Au with Ag); 1/10 gold without silver-intensification (1/10 Au only) and PBS solution (PBS treatment only).

PCA was employed to see how the enhanced spectra clustered compared to the control (Figure 5). Unsurprisingly, the samples with 1/10 silver-intensified gold nanoparticles clusters furthest away from the control, the samples with 1/100 silver-intensified gold nanoparticles shows some separation from the control and the samples with just 1/10 cationic gold overlaps with the control. The different sample classes' ellipsoid boundaries indicate the 95% confidence limits. Clearly the cluster for 1/10 silver-intensified gold nanoparticles shows no overlap with the control.

**Figure 6:**
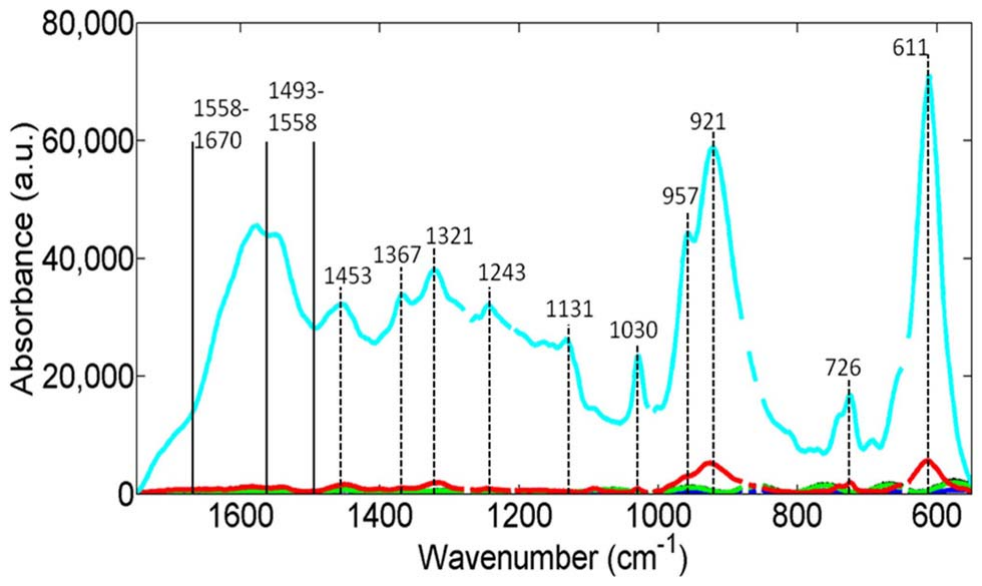
Mean spectra (light blue) of the 1/10 silver-intensified gold samples. Showing the spectral peaks which demonstrate the highest levels of SERS-enhancement. The pack with the highest level of enhancement is at 611 cm^−1^.

**Figure 7:**
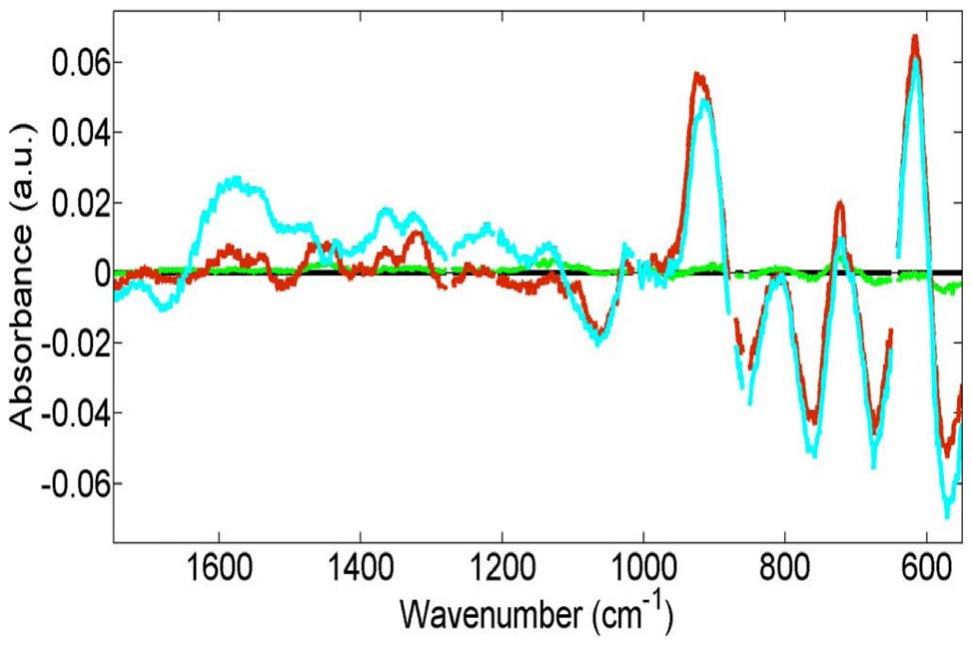
The PCA cluster vectors with the PBS-treated control samples set as the origin. The green plot consists of 1/10 gold only samples deviates only slightly from the origin, but both plots from the 1/100 silver-intensified gold (red) and the 1/10 silver-intensified gold (light blue) samples are surprisingly similar over the 1000–600 cm^−1^ spectral regions. Both plots show maximum deflection from the origin around the 611 cm^−1^ spectral region.

**Figure 8:**
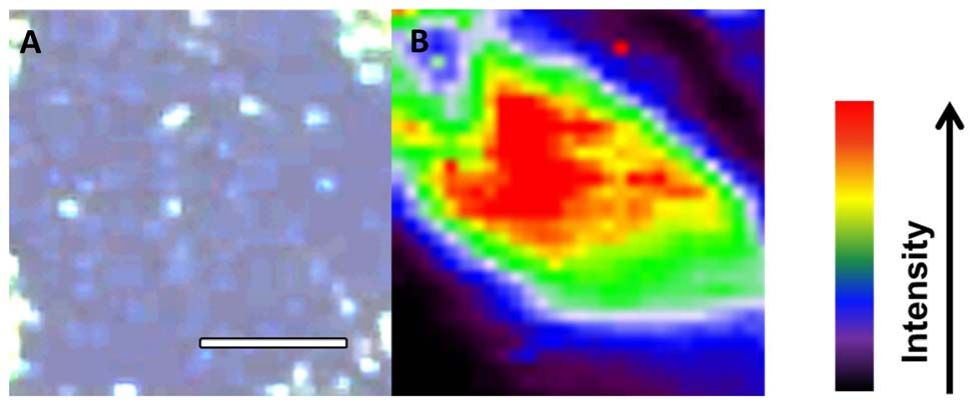
Light micrograph of endothelium. Endothelial cell treated with 1/10 silver-intensified gold nanoparticles (**A**). Scale bar  = 10 µm. SERS-enhanced spectral map of the same region taken at a wavenumber of 611 cm^−1^ (**B**).

From the mean spectra, there are several peaks or regions within the spectra that are readily identifiable (Figure 6); these are highlighted in Table 1. The cluster vectors shown in Figure 7 show relevant peaks from each group compared to each other *via* a vector taken from a central point, in this case it is the PBS control group. The groups with 1/10 silver-intensified gold and 1/100 silver-intensified gold were the only groups to show significant peaks whilst the other groups had peaks that were not above the set threshold value of significance. Interestingly, the plot from the 1/100 silver-intensified gold is very similar to the 1/10 silver-intensified gold over the 1000–600 cm^−1^ spectral region, although the degree of SERS-enhancement is much less. Following on from this, a SERS-enhanced spectral map at 611 cm^−1^ is shown (Figure 8). Interestingly, the map shows a non-uniform signal distribution for this wavenumber over the cell surface.

**Table 1:** Tentative peak assignations for the most enhanced peaks of the spectra.

Wavenumber (cm^−1^)	Tentative peak assignment	Reference
608	Cholesterol	[27], [28]
614	Cholesterol ester	[27], [28]
618	C-C protein twist	[28], [29]
726	C-S protein CH_2_ rocking	[28], [30]
920	C-C stretch of proline ring/glucose/lactic acid	[28], [29]
957	Hydroxyapatite/carotenoid/cholesterol	[28], [30]
1030/1031	Phenylalanine	[28], [29]
1131	Fatty acid	[27], [28]
1243	Amide III	[28], [30]
1321	Amide III (α helix)	[28]
1367	CH_3_ symmetric stretching in lipids	[28], [31]
1453	Protein band; C-H stretching	[28]
1493–1558	Amide II and various amino acids	[28], [29]
1558–1670	Amide carbonyl group vibrations	[28]

## Discussion

Our primary aim in this investigation was to determine if it is feasible to use silver-intensified cationic gold nanoparticles to obtain SERS-enhanced spectra from the cell surface. The protocol we have used has proved effective in covering the surface of the endothelial cells with silver-intensified gold nanoparticles (Figure 1). Our methodology produces what appears to be an even distribution of nanoparticles over the cell surface, indicating that the surface charge to which the cationic gold attaches is fairly uniform over the surface of the cells (Figures 2 and 3). This is consistent with previous work on the glycocalyx of the cell surface.

Silver-intensification, also known as silver-enhancement, is widely used in electron microscopy. The intensification solution consists of silver ions and a reducing agent which results in the growth of a metallic silver shell around the gold particles [2]. A recent study has characterized the silver nanoparticles resulting from several commercially available intensification solutions including the one used in this paper (see Methods), showing that the growth of the metallic silver nanoparticle is at a rate of 41 nm per minute at room temperature [2]. This is broadly in line with our findings of a modal diameter of 1100 nm after 20 minutes. Almost certainly some of the particles measured in this study are aggregates accounting for our modal size of 1100 nm rather than the predicted modal size of 820 nm based on linear growth of 41 nm/minute [2]. This is supported by the SEM images of the nanoparticles (Figure 2D) which appear to show that some of the nanoparticles are aggregates.

The effect of the silver-intensified gold nanoparticles has been to produce a 100-fold increase in signal intensity (Figure 4). The SERS-enhanced spectra of both the 1/10 and 1/100 groups are significantly enhanced compared to the control (Figure 5). However, the degree of enhancement is much greater with the 1/10 silver-intensified gold group. This SERS-enhancement occurs across the entire biomolecular range. Previous work shows that the maximum SERS-enhancement effect will be derived from the immediate environment next to the nanoparticles, within a few 10's of nanometers rather than from the whole cell as would occur with conventional Raman spectroscopy [23]. This means that the majority of the SERS signal must be coming from the endothelial cell membrane, and therefore should contain specific information about the biomolecular composition of the cell membrane. Analysis of the spectra supports this, as we found that all of our enhanced peaks can be assigned to biomolecules found within the plasma membrane (Figure 6; Table 1). These wavenumbers can be assigned [27]–[31] to a variety of proteins and lipids, which are all consistent with the composition of the cell membrane.

As shown in Figure 1, the endothelial plasma membrane is composed of a lipid bilayer consisting of phospholipids with long fatty acid chains containing both saturated and unsaturated bonds. The cell membrane contains large amounts of cholesterol. Dissolved or embedded within the lipid bilayer are numerous transmembrane proteins with hydrophobic α-helical structures “dissolved” within the lipid bilayer itself and β-sheet structures exposed to the hydrophilic environment on either side of the membrane. Also, present on the cell surface are numerous glycol-lipids and glycoproteins. The glycocalyx of endothelial cells is made up from the carbohydrate chains on the surface of the cell and can project several hundred nanometers above the cell membrane [32]. Many components of the glycocalyx are negatively charged, *e.g.*, the glycosaminoglycan chains known to be present on the endothelial surface [1].

Figures 6 and 7 show the SERS-enhanced peaks, while Table 1 shows that these can be assigned to a variety of lipids and proteins. All of the wavenumber assignments are consistent with being derived from membrane-associated components. The plasma membrane contains many different types of lipids with both integral and peripheral membrane proteins. The plasma membrane itself is only about 9 nm thick. As mentioned earlier, previous work suggests that the SERS effect only occurs very close to the silver nanoparticles [3], [23], probably less than 10 nm. This means that pretty much all of the enhanced signal will be derived from the plasma membrane.

As part of this project we chose the region of the biomolecular spectra which was most enhanced. This peak centers on the wavenumber 611 cm^−1^, which covers the cholesterol regions (608 cm^−1^ to 614 cm^−1^). Cholesterol is a major component of animal cell membranes varying between often making up more than 25% of the membrane composition. Interestingly, the spectral map of this wavenumber shows there is high signal intensity in the center of the cell. One possible, speculative, explanation for this is that we are seeing the presence of lipid rafts. Lipid rafts are microdomains of the plasma membrane which have a different composition to other regions of the plasma membrane [24], [25]; they are known to have a different lipid composition to the rest of the membrane and reports suggest that the cholesterol concentration is much higher than in the rest of the plasma membrane [33]. Some reports suggest that lipid rafts may be composed of 50% cholesterol [34]. The existence of lipid rafts is still slightly controversial and none have yet been reported on corneal endothelial cells. However, the spectral map we have obtained would support the existence of lipid rafts on the corneal endothelial cells. Although the central area is larger than most reported lipid rafts, we speculate that these might be groups of lipid rafts too small to be resolved with our mapping resolution. There are also smaller domains, which are within the reported size of lipid rafts.

In conclusion, this work has shown that it is possible to get a 100-fold signal enhancement in cell surface spectra with silver-intensified cationic gold. The cationic gold attaches evenly to the cell glycocalyx and this methodology is useful for the study of the plasma membrane. The results have provided some possible evidence for the heterogeneous distribution of membrane components on corneal endothelial cells. This method does not require the use of antibodies, antibodies have both benefits and disadvantages; they allow a more targeted approach bringing the nanoparticles to a specific epitope on the cells surface but the procedure is more complex and time-consuming and there have been some concerns expressed as to what the contribution of the antibody might be to the spectra. Our study demonstrates an approach that has produced a 100-fold SERS-enhancement of the spectral signal which appears to be derived chiefly or solely from the endothelial cell membrane. Additionally, spectral mapping has provided possible evidence on the heterogeneity of some of the plasma membrane components.
